# A 60-Year-Old Woman With Endometrial Intraepithelial Neoplasia: A Case of Persistent Postmenopausal Bleeding

**DOI:** 10.7759/cureus.103797

**Published:** 2026-02-17

**Authors:** Marissa Wu, Harkirat Kaur, Sorivel Sosa

**Affiliations:** 1 Research, Derryfield School, Bedford, USA; 2 Research Department, Global Health Leaders, Bend, USA; 3 Research, University of Washington, Bothell, USA

**Keywords:** atypical endometrial hyperplasia, clinical case report, endometrial intraepithelial neoplasia (ein), hysterectomy, postmenopausal bleeding

## Abstract

Endometrial intraepithelial neoplasia (EIN) is a premalignant lesion of the endometrium that may progress to endometrioid adenocarcinoma. This case is unique because EIN occurred in a 60-year-old woman who was 10 years postmenopausal, lacked risk factors, and had symptoms despite prior treatment for a presumed benign endometrial polyp.

The patient presented with several months of pelvic pain and postmenopausal spotting. Laboratory studies were largely normal. Transvaginal ultrasound revealed a slightly thickened 7-mm endometrium and multiple intramural fibroids, suggesting benign pathology.

Due to continued bleeding, an endometrial biopsy was performed and demonstrated atypical hyperplasia consistent with EIN. Given the patient’s postmenopausal status and the high risk of progression to carcinoma, definitive treatment with hysterectomy was recommended.

This case highlights the importance of continued evaluation of persistent postmenopausal bleeding, even when initial findings suggest benign disease. Early biopsy and clinical vigilance are essential to prevent progression to endometrial cancer.

## Introduction

Endometrial intraepithelial neoplasia (EIN) is a precancerous condition that forms in the glands of the uterine lining and can develop into endometrioid-type endometrial cancer. EIN is a rare disease with about 44 cases per 100,000 people worldwide [1]. It is unique because it represents a monoclonal premalignant lesion that directly precedes endometrioid endometrial adenocarcinoma, making it a clearly identifiable precursor in the carcinogenic pathway [1]. EIN is characterized by the presence of latent, genetically mutated glands that may appear morphologically normal for years before pathological changes become evident [1].

Reliable data on EIN are limited in the Dominican Republic, but national and global cancer registry data provide insight into the broader uterine cancer burden. According to the International Agency for Research on Cancer (2022), uterine corpus cancers ranked 16th in overall incidence among Dominican women, accounting for roughly 209 new cases in 2022 and a cumulative lifetime risk of 0.42% [2]. Most endometrial malignancies arise in postmenopausal women, with incidence peaking between 55 and 70 years of age [3]. Although country-specific prevalence data for EIN are unavailable, patterns from comparable regions suggest that perimenopausal and early postmenopausal women face the highest risk, particularly when exposed to unopposed estrogen. Globally, obesity, hypertension, diabetes, and prolonged estrogen exposure are well-established risk factors that likely contribute to the observed age-related and hormonal distribution in the Dominican population [3].

EIN is a precancerous lesion that can be difficult to distinguish from benign hyperplasia or early carcinoma. Its identification requires nuanced histopathologic criteria and is often under-recognized. This low incidence heightens the importance of identifying atypical or early presentation promptly to ensure timely and effective intervention. EIN often manifests through vague or mild symptoms like irregular uterine bleeding, which can easily be overlooked or misdiagnosed, particularly in younger or premenopausal individuals. While not all cases of EIN advance to carcinoma, the likelihood of progression is markedly higher than in benign endometrial conditions, with research indicating a 45- fold increased risk of developing cancer [1]. Accurate diagnosis of EIN versus other forms of hyperplasia is essential for guiding treatment. The systematic review emphasizes the importance of early detection and appropriate surveillance to prevent progression to endometrial carcinoma [3].

## Case presentation

A 60-year-old Dominican woman with a history of EIN was evaluated. She reported normal female pubertal development, regular menstrual cycles prior to menopause, and external genitalia appropriate for age and sex. Her sexual debut occurred at 20 years of age. She was postmenopausal for approximately 10 years and presented to gynecologic care with pelvic pain and several months of light, intermittent spotting-type vaginal bleeding. During prior outpatient evaluations, she had been diagnosed with an endometrial polyp and treated accordingly; however, both vaginal bleeding and pelvic pain persisted.

The physical examination was largely unremarkable. The patient appeared in good general condition, with no palpable masses in the head or neck and no lymphadenopathy. Cardiopulmonary examination was normal. Breast examination revealed asymmetry without palpable masses. The abdomen was soft and non-tender, and the external genital examination was appropriate for age. Rectovaginal examination demonstrated a short vaginal length of approximately 2 cm and a rectal ampulla filled with fecal material, limiting complete evaluation of the parametrium. The extremities were symmetrical with intact peripheral pulses, no edema, and an old surgical scar noted on the right lower extremity. Laboratory evaluation showed normal complete blood count, coagulation profile, and metabolic parameters, with the exception of albuminuria on urinalysis. Viral screening tests were negative.

**Figure 1:**
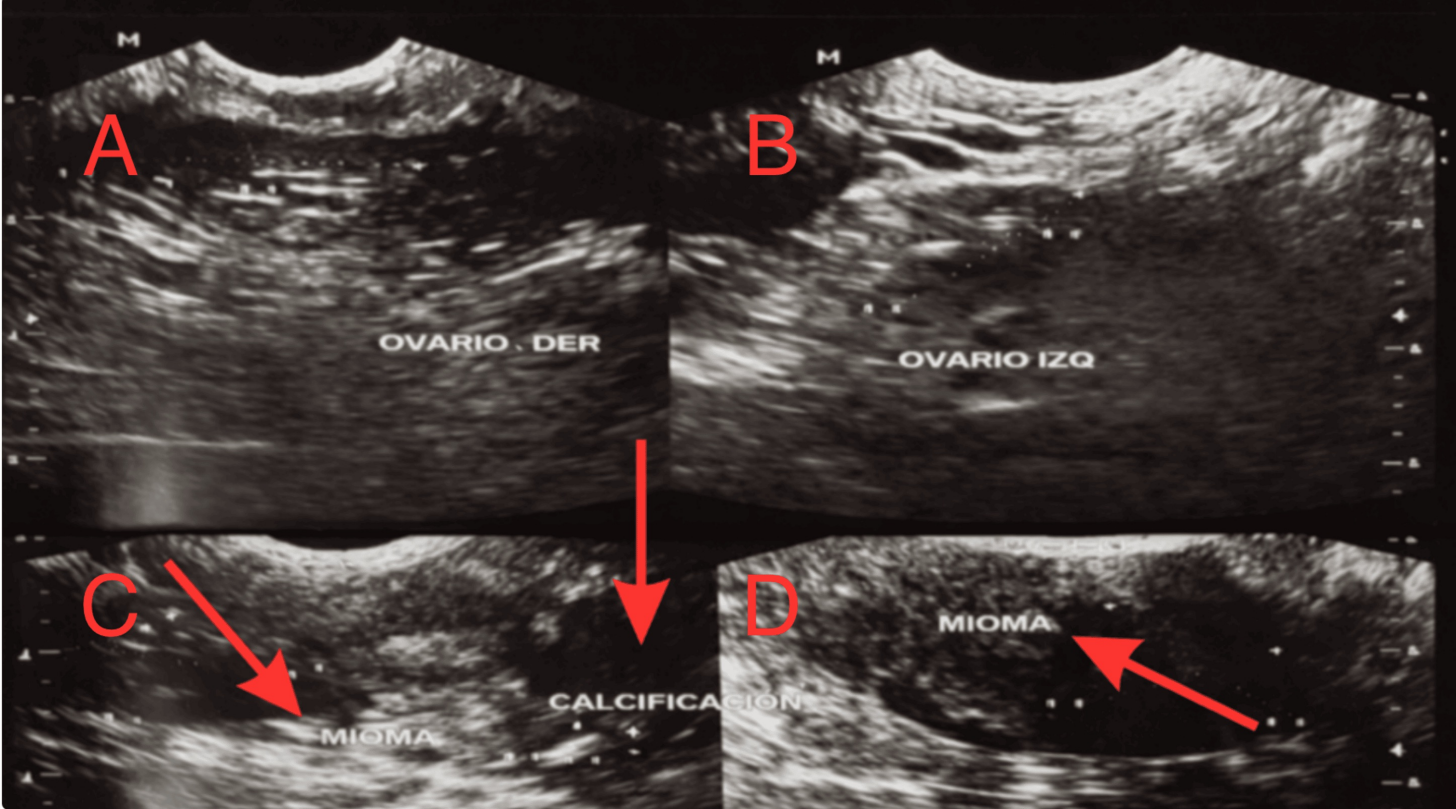
Transvaginal ultrasound findings of the uterus and adnexa (left to right). (A) Right ovary with normal size and echotexture, without focal masses. (B) Left ovary with normal sonographic appearance and no adnexal lesions. (C) Intramural uterine fibroid (leiomyoma) with calcification, appearing as a hyperechoic focus with posterior acoustic shadowing. (D) Additional intramural uterine fibroid demonstrating a solid, well-circumscribed appearance. These benign imaging findings contributed to diagnostic overlap and delayed recognition of underlying endometrial pathology in this postmenopausal patient.

**Figure 2:**
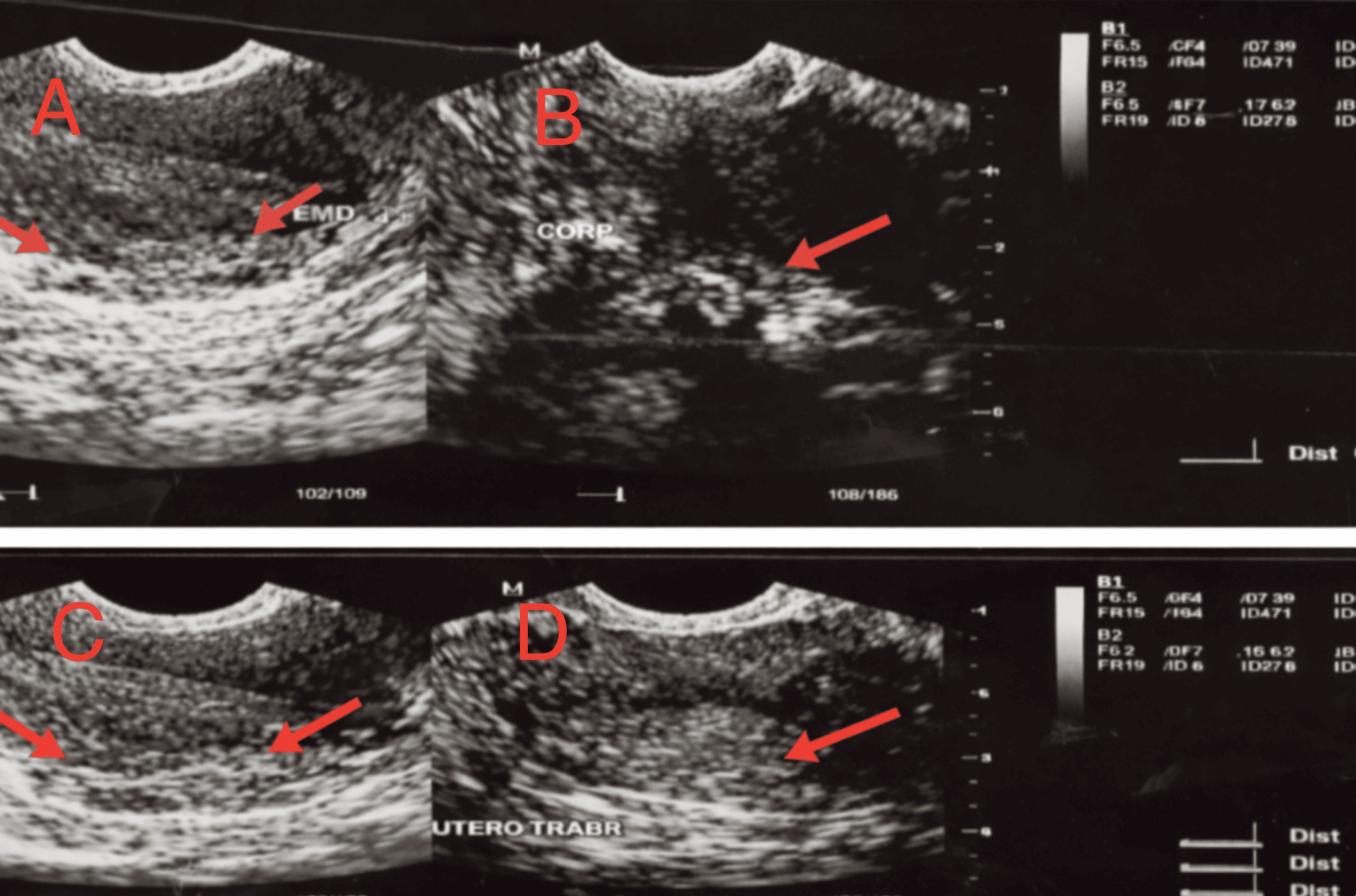
Transvaginal ultrasound evaluation of the uterus and endometrium (left to right) (A) Longitudinal view of the uterus demonstrating the endometrial stripe with measured thickness of approximately 0.76 cm. (B) Uterine corpus showing heterogeneous myometrial echotexture without focal mass. (C) Longitudinal uterine view highlighting the endometrium and surrounding myometrium. (D) Trabeculated appearance of the uterine myometrium, consistent with chronic uterine changes. These findings demonstrate mild endometrial thickening and myometrial abnormalities that, while nonspecific, warranted further evaluation in the setting of persistent postmenopausal bleeding.

Doppler ultrasound of the lower extremities revealed mild arteriosclerosis and venous insufficiency in the right leg. Transvaginal ultrasonography demonstrated a mildly thickened endometrium measuring approximately 7 mm and multiple intramural uterine fibroids, including one with calcification, as shown in Figures 1, 2. Both ovaries appeared normal. Given the persistence of postmenopausal bleeding despite previously reported benign findings, an endometrial biopsy was performed. Histopathological evaluation revealed atypical endometrial hyperplasia consistent with EIN, confirming a premalignant condition.

The diagnostic workup integrated clinical presentation, laboratory findings, and imaging results to elucidate the cause of the patient’s persistent postmenopausal bleeding. Although uterine fibroids and a previously diagnosed endometrial polyp initially suggested benign etiologies, the continuation of symptoms warranted further investigation. Transvaginal ultrasound demonstrated only mild endometrial thickening, prompting tissue sampling. The endometrial biopsy confirmed hyperplasia with atypia consistent with EIN. Given the substantial risk of concurrent or subsequent endometrial carcinoma associated with EIN, this finding underscored the need for prompt and definitive management to prevent malignant progression.

In light of the patient’s postmenopausal status and the established risk of progression or coexistence of endometrial carcinoma in patients with EIN, definitive surgical management with hysterectomy was recommended in accordance with current clinical guidelines. Throughout follow-up, the patient remained clinically stable, with no unexpected events reported. Post-diagnostic evaluation emphasized the importance of timely intervention to prevent progression to invasive carcinoma, and the patient demonstrated full adherence to the proposed treatment plan.

The definitive treatment recommended was hysterectomy, given the diagnosis of atypical endometrial hyperplasia and the associated risk of progression to invasive disease. The patient’s previous surgical history included tibia fracture repair, which was not directly related to her current gynecological condition. In addition, preventive gynecological monitoring such as biopsy and ultrasound was conducted to enable early detection of endometrial pathology. A uterus test was conducted on 08/01/2024 and a two-dimensional arterial and venous study of the lower extremity was performed on 09/24/2024. Initial pharmacologic treatment for the polyp and associated bleeding proved insufficient, as symptoms persisted. Consequently, outpatient pharmacologic therapy was discontinued once it failed to control bleeding and pain. Despite this, preventive interventions, including routine check-ups and laboratory monitoring, continued throughout the patient’s clinical course. Ultimately, transition to surgical management was indicated based on biopsy findings of atypical endometrial hyperplasia and the risk of progression to malignancy. This justified escalation to definitive surgical intervention.

During follow-up, the patient reported persistent light bleeding and pelvic discomfort despite earlier treatment for a presumed endometrial polyp, prompting further evaluation. Clinicians monitored her progress through repeat ultrasound imaging, Doppler studies, and laboratory testing, which remained largely within normal limits aside from positive albumin. The patient adhered to all recommended diagnostic procedures, including biopsy, which she tolerated without complications. The histopathology confirming atypical hyperplasia/EIN guided the decision for definitive management with hysterectomy, consistent with clinical guidelines for postmenopausal patients with premalignant endometrial disease. Throughout follow-up, no unexpected events were recorded, and the patient remained clinically stable. Post-diagnostic assessments emphasized the importance of timely intervention to prevent progression to invasive carcinoma, and the patient demonstrated full compliance with the treatment plan.

## Discussion

This case is particularly unusual because the patient was 10 years postmenopausal, had persistent bleeding despite prior treatment for a presumed benign polyp, and lacked common risk factors such as obesity, diabetes, or prolonged estrogen exposure. EIN lesions share molecular features with endometrial cancer, and progression occurs through the accumulation of additional genetic mutations, a process reflected in this patient’s atypical presentation. The combination of delayed postmenopausal onset, a prior benign diagnosis, and ongoing symptoms makes this a distinctive example of the diagnostic challenges associated with EIN. A reasonable hypothesis for the clinical decision to proceed directly to hysterectomy, rather than performing a second biopsy, is that the persistence of bleeding in a postmenopausal patient despite prior intervention raised sufficient concern for underlying carcinoma to justify definitive management without further delay. This approach aligns with evidence showing that postmenopausal women with biopsy-proven EIN have a high likelihood of concurrent malignancy and benefit from timely surgical treatment.

Published literature provides context for understanding the diagnostic and epidemiologic relevance of this case. EIN is described as a clonal premalignant lesion associated with a 30-50% risk of concurrent endometrial carcinoma, supporting the rationale for definitive surgical management in this patient [1]. Progression from EIN to endometrial carcinoma occurs most in postmenopausal women and may develop over four years, with carcinoma detected in 30-45% of women at the time of EIN diagnosis [3]. Endometrial cancer is rising in the Dominican Republic, where limited cancer surveillance may contribute to insufficient detection and the burden of uterine cancer is also increasing among Hispanic and Latina women in the United States [2,4]. Patterns among Caribbean-origin Hispanic women note that they have the highest proportions of advanced-stage disease, suggesting disparities in disease risk, detection, and access to care or public health [5]. Early imaging and endometrial biopsy may also fail to detect EIN because the lesion can be patchy and focal, making it susceptible to sampling error during blind biopsy procedures. Reliance on endometrial thickness thresholds in postmenopausal women may not reliably exclude premalignant disease [3]. The patient’s Dominican background and delayed identification of the lesion reflect these broader population trends and the significance of public health. This case mirrors patterns described in literature, emphasizing the importance of persistent evaluation, early biopsy, and timely treatment to prevent malignant progression.

A major strength of this case was the comprehensive diagnostic approach used to evaluate persistent postmenopausal bleeding, but its true novelty lies in the identification of EIN in a patient without classic risk factors, abnormal imaging, or concerning laboratory findings. Despite an initial benign diagnosis and reassuring studies, the persistence of symptoms prompted continued investigation, highlighting an atypical presentation that could easily have been overlooked in routine practice. This case demonstrates how EIN can emerge even when clinical, demographic, and laboratory indicators do not align with the expected risk profile, underscoring the importance of clinical vigilance beyond guideline‑listed risk factors. Although advanced molecular testing such as PTEN or PAX2 analysis was not performed, histopathology provided a definitive diagnosis, consistent with current standards [1]. Overall, this case contributes to existing literature by illustrating a subtle, non‑classic presentation of EIN and reinforces the need for persistent evaluation of postmenopausal bleeding even when preliminary findings appear benign.

## Conclusions

This case underscores how persistent postmenopausal bleeding warrants thorough evaluation, as benign findings can mask premalignant disease such as EIN. The patient’s biopsy‑confirmed EIN, combined with evidence showing a substantial risk of concurrent or future carcinoma in postmenopausal women, supported the decision for definitive surgical management. Early biopsy and timely hysterectomy align with current clinical guidelines and are associated with improved outcomes, reinforcing the importance of vigilant follow‑up in gynecologic care. From a public health perspective, increasing awareness of EIN and promoting prompt assessment of abnormal bleeding can reduce delays in diagnosis, prevent progression to invasive cancer, and ultimately improve population-level survival.
